# Perceptual Doping: A Hypothesis on How Early Audiovisual Speech Stimulation Enhances Subsequent Auditory Speech Processing

**DOI:** 10.3390/brainsci13040601

**Published:** 2023-04-01

**Authors:** Shahram Moradi, Jerker Rönnberg

**Affiliations:** 1 Department of Health, Social and Welfare Studies, Faculty of Health and Social Sciences, University of South-Eastern Norway, 3918 Porsgrunn, Norway; 2 Department of Behavioral Sciences and Learning, Linnaeus Centre Head, Linköping University, 581 83 Linköping, Sweden

**Keywords:** audiovisual speech training, audio speech training, perceptual doping, hearing loss, auditory speech identification, Ease of Language Understanding Model (the ELU model), cognitive function

## Abstract

Face-to-face communication is one of the most common means of communication in daily life. We benefit from both auditory and visual speech signals that lead to better language understanding. People prefer face-to-face communication when access to auditory speech cues is limited because of background noise in the surrounding environment or in the case of hearing impairment. We demonstrated that an early, short period of exposure to audiovisual speech stimuli facilitates subsequent auditory processing of speech stimuli for correct identification, but early auditory exposure does not. We called this effect “perceptual doping” as an early audiovisual speech stimulation dopes or recalibrates auditory phonological and lexical maps in the mental lexicon in a way that results in better processing of auditory speech signals for correct identification. This short opinion paper provides an overview of perceptual doping and how it differs from similar auditory perceptual aftereffects following exposure to audiovisual speech materials, its underlying cognitive mechanism, and its potential usefulness in the aural rehabilitation of people with hearing difficulties.

## 1. Introduction

Language understanding is fundamentally a multisensory process, as face-to-face communication in humans aids language understanding by providing not only verbal cues but also non-verbal cues such as emotional expression, body gesture, and facial expression. The importance of face-to-face interactions is evident in adverse listening conditions when access to verbal cues is limited because of external noise or hearing loss.

Sumby and Pollack [1] were the first to show that the addition of visual speech cues to an auditory speech signal enhances speech intelligibility, especially in noisy conditions. Subsequent studies demonstrated the advantage of audiovisual speech perception over audio-only speech perception when perceiving speech signals in degraded listening conditions [2,3]. Furthermore, more recent studies revealed that the addition of visual speech cues to an auditory speech signal reduces the cognitive demands required to process the speech signals in noisy listening conditions, both for normal hearing listeners [4] and for people with hearing loss [5].

In addition, studies have also found audiovisual aftereffects following exposure to audiovisual speech materials [6,7]. Bertelson et al. [6] found that prior stimulation to a video of a face articulating /ada/ or /aba/ accompanied by an auditory ambiguous sound halfway between /d/ and /b/ (A?Vd or A?Vb) caused a phonetic recalibration effect on subsequent auditory ambiguous sound perception. Those who were first exposed to A?Vd subsequently perceived ambiguous auditory sounds halfway between /d/ and /b/, often as /d/, and those who were first exposed to A?Vb subsequently perceived ambiguous auditory sounds mainly as /b/. Such a phonetic recalibration effect was not observed after exposure to congruent unambiguous AbVb or AdVd conditions. Bertelson et al. [6] reasoned that early exposure to incongruent A?Vd or A?Vb speech stimuli biased or recalibrated subsequent auditory ambiguous speech tokens in favor of visual components of a prior incongruent audiovisual speech signal.

We found a specific audiovisual facilitation effect on subsequent auditory speech-processing tasks. Moradi et al. [4], in a between-subject experimental study, found that participants exposed to gated audiovisual consonants, words, and sentence-final word identification tasks performed better on a subsequent auditory sentence identification in noise task (Hearing in Noise Test [HINT] [8]) than those exposed only to auditory consonants, words, and sentence-final word identification tasks.

In a randomized control study, Lidestam et al. [9] divided normal hearing participants into three groups: (1) a group exposed to gated audiovisual consonants and words, (2) a group exposed to gated auditory consonants and words, and (3) a control group who only watched a video clip. The HINT scores for each group were recorded before and after exposure to the gated audiovisual stimuli, auditory stimuli, or video clip. Only the group exposed to the gated audiovisual consonants and words subsequently performed better on the HINT, not the other two groups.

Using the same gated audiovisual and auditory gated speech stimuli as employed by Lidestam et al. [9], Moradi et al. [5] studied the efficiency and maintenance of gated audiovisual speech training on auditory HINT performance in elderly hearing-aid users. The results showed that gated audiovisual speech stimulation resulted in better performance on the HINT. Importantly, the audiovisual training effect on HINT performance was maintained after one month.

Finally, using data from the n200 study [10], which comprised 200 hearing-impaired hearing-aid users, Moradi et al. [11] found that prior audiovisual speech stimulation generated larger benefits over auditory speech stimulation in terms of the subsequent processing of auditory speech stimuli for the correct identification of consonants and vowels and the correct discrimination of vowel durations.

We have dubbed this rapid type of perceptual learning “perceptual doping”, arguing that even a short exposure to audiovisual speech stimuli recalibrates or retunes phonological and semantic processing maps in semantic long-term memory in a way that facilitates subsequent auditory processing of speech stimuli for correct identification (Moradi et al. [5,11]).

This audiovisual facilitation effect on subsequent auditory speech processing cannot be explained by concepts like perceptual learning only or using lexical knowledge to learn how to categorize speech sounds [12]. Perceptual learning reflects enhanced performance in a task achieved via repeated stimulation of that task. In Lidestam et al. [9] and in Moradi et al. [4,5], the enhancement effect on auditory sentence-in-noise identification was only observed after exposure to audiovisual speech materials and not after auditory stimulation alone. In addition, the materials used in prior audiovisual speech exposure were consonants and words, while the outcome auditory task was sentence-in-noise identification. Furthermore, the talkers in the prior audiovisual exposure and subsequent auditory sentence-in-noise identification tasks were different. Norris et al. [12] showed that listeners benefit from their lexical knowledge in the perceptual learning process to interpret ambiguous speech sounds. Assuming a perceptual doping notion, the recalibrated phonological and lexical maps following exposure to a congruent audiovisual speech signal help listeners to better identify subsequent auditory speech signals. So, the benefit from existing lexical knowledge for subsequent auditory speech identification is not the main point in the perceptual doping notion. We did not examine the extent to which lexical knowledge impacts the benefit provided by prior audiovisual speech stimulation on the subsequent processing of auditory speech signal for correct identification. Van Linden and Vroomen [7] showed that both visual speech cues and lexical knowledge play a similar role in the phonetic recalibration effect. Future studies are needed to evaluate the benefit provided by lexical knowledge and prior audiovisual speech stimulation on the subsequent processing of auditory speech signals. Further, the perceptual doping notion also means that the locus of the effect cannot be ascribed to repetition priming or “pop out” ([13]). We argued that the better task performance in subsequent auditory sentence-in-noise identification was merely the result of prior exposure to audiovisual speech materials that retuned phonological and lexical maps more distinct, with sharp boundaries, and easily accessible, consequently easing the subsequent mapping of auditory speech input onto phonological and lexical items during sentence-in-noise identification.

Here, we reason that the perceptual doping notion differs from the audiovisual phonetic recalibration effect. First, the audiovisual speech materials in our prior research were different from the work by Bertelson et al. [6]. The audiovisual speech stimuli in Moradi et al. [4,5] and Lidestam et al. [9] were congruent but degraded by a speech signal (background noise) that was presented to the participants in a gating format. In Moradi et al. [11], speech items were auditory and audiovisual consonants and vowels that were presented to the participants in silence in a gating format. A facilitation effect (i.e., perceptual doping) was observed only after exposure to audiovisual speech items but not to auditory ones. Second, theoretical assumptions of the perceptual doping notion are different from the audiovisual phonetic recalibration. According to the perceptual doping hypothesis, simple exposure to audiovisual speech stimuli retunes phonological and lexical maps that subsequently facilitate auditory processing of speech signals for correct identification. On the other hand, audiovisual phonetic recalibration assumes a recalibration of an existing phonetic representation by shifting the subsequent ambiguous auditory speech sound toward the visual component of a prior incongruent and ambiguous audiovisual speech signal. In short, we reason that the congruency of audiovisual speech signals differentiates the perceptual doping notion from the phonetic recalibration effect.

## 2. Cognitive Mechanism behind the Perceptual Doping Phenomenon

The perceptual doping effect can be understood in terms of the Ease of Language Understanding (ELU) model. In particular, within the ELU framework, there is a perceptual-linguistic component that assumes a Rapid, Automatic, Multimodal Binding of PHOnological information (RAMBPHO [14,15,16]). RAMBPHO serves as an input buffer that binds, integrates, and processes multimodal input in order to map it with corresponding phonological and lexical representations in semantic long-term memory. In fact, RAMBPHO is the default mode for the implicit processing of speech signals that directly and implicitly unlock the multimodal phonological features of speech signals for accurate mapping onto phonological and lexical representations in semantic long-term memory. This default mode of processing incoming speech signals takes place during the first 100–400 ms of the speech signals being presented [15]. We speculate that the initial audiovisual speech stimulation recalibrates the default mode of processing speech signals in the RAMBPHO input buffer such that congruent auditory and visual speech signals reduce the uncertainty of speech input, particularly in degraded listening conditions. In fact, van Wassenhove et al. [17] found that visual speech cues have a predictive role for the auditory component of a congruent audiovisual speech signal for identification. They reported that visual speech cues speed up the neural processing of congruent auditory input during the first 100 ms of the audiovisual speech signal being presented. In addition, Zion-Golumic et al. [18] revealed that audiovisual over auditory speech presentation results in an enhanced capacity of the auditory cortex to process the temporal features of speech signals in degraded listening conditions. Further, Mégevand et al. [19], in a study of the electrical activity of the human brain via implanted electrodes, found that visual speech cues in an audiovisual speech signal improved phase-tracking and reduced the amplitude of evoked responses to congruent auditory speech signals. Frei et al. [20] also reported that visual speech cues increase neural tracking of the speech cues, particularly in the right auditory clusters, which subsequently results in better speech in noise comprehension in older adults with hearing loss. We speculate that after audiovisual speech stimulation, the improved capacity of the auditory cortex to track down the most critical features of speech, particularly in degraded listening conditions, does not vanish and is expected to persist for a longer period.

## 3. Perceptual Doping and Aural Rehabilitation of People with Hearing Difficulties

Most recent studies used auditory (and cognitive) training to enhance speech intelligibility in people with hearing loss. Stropahl et al. [21], in their review of auditory training for improving speech processing skills in people with hearing loss, concluded that intense auditory training might enhance non-trained auditory speech tasks in those with hearing loss.

However, the maintenance of auditory (and cognitive) training effects on auditory speech tasks requires further research. To our knowledge, only a few studies have investigated the efficiency of audiovisual speech training on subsequent auditory speech processing tasks. As mentioned above, Moradi et al. [5] showed that only a short-term exposure (around 30–40 min) to gated audiovisual consonants and words for identification resulted in better auditory performance in the HINT, a non-trained task, in elderly hearing-aid users. In addition, the positive effect of audiovisual speech stimulation remained after one month of training. Rao et al. [22] studied the effect of ReadMyQuips™ (RMQ), an audiovisual training program, on HINT performance in elderly people with hearing loss. The results showed that RMQ improved HINT scores in the experimental group that participated in the RMQ training program.

Tye-Murray et al. [23] studied auditory and audiovisual speech training to listening to noise and speech-reading performance in children with hearing loss. The results showed that both auditory and audiovisual speech training improved both listening and speech-reading performance in children with hearing loss. In addition, the effect of auditory training was more evident in the listening performance, while the effect of audiovisual speech training was evident for both listening and speech-reading performance.

Sato et al. [24] studied the feasibility of in-home audiovisual speech training using a tablet computer to improve speech intelligibility in people with hearing loss. The participants used either a hearing aid or a cochlear implant. The participants listened to audiovisual monosyllable words that were spoken by a female talker. The training took 3 months, and speech intelligibility was recorded for untrained words, trained words, and monosyllables. Results showed that audiovisual speech training using a tablet computer improved untrained and trained words after the 3-month training with audiovisual speech stimuli.

We reason that the addition of visual cues to auditory (and cognitive) training speech materials boosts the effectiveness of aural rehabilitation programs for people with hearing loss in terms of both efficiency and the maintenance of training effects on the listening capacities of persons with hearing loss. This speculation requires further investigation; future studies should consider the transfer of the learning effect from trained to non-trained speech materials, subjective hearing satisfaction in daily life, cognitive function in people with hearing loss, and the maintenance of training effects.

### 3.1. Perceptual Doping and Controlling Experimental Setup for Collecting Auditory and Audiovisual Speech Data

As prior audiovisual speech exposure boosts the subsequent auditory identification of speech stimuli, caution should be taken when collecting auditory and audiovisual speech data in within-subjects studies when the modality of presentation (auditory or audiovisual) is randomized across participants. In addition, caution should be taken if the participants are first tested in an audiovisual and then an auditory modality. In fact, the perceptual doping may cause a type II error (in failing to reject a false null hypothesis) in a within-subjects design, which may result in non-significant differences between auditory and audiovisual speech data or even lead to better performance of auditory speech stimuli than audiovisual stimuli. We suggest that future studies adopting a within-subjects experimental design consider collecting data using a fixed-order presentation (instead of a randomized-order presentation) by collecting auditory speech data first and then audiovisual speech data. Nevertheless, although a between-subject experimental design can be used to control perceptual doping and priming effects, individual differences across participants (e.g., differences in terms of speech-reading ability, auditory acuity, and audio and video integration ability) are methodological disadvantages that should be taken into account when comparing auditory and audiovisual groups.

### 3.2. Suggestions for Future Studies

(1) The extent to which a short period of exposure to audiovisual speech stimuli facilitates visual-only and/or audiovisual speech processing for correct identification is an interesting research topic for future studies. Knowledge is scarce concerning how audiovisual speech stimulation can enhance the processing of visual-only speech cues for correct identification, particularly in people with hearing loss. In face-to-face communication, people with hearing difficulties rely more on visual speech cues, as access to the auditory component of audiovisual speech signals is limited by background noise or hearing loss.
(2) The extent to which audiovisual speech training can improve cognitive function is another interesting research topic for future studies. Fergusson and colleagues [25,26] were the first to show that auditory training can improve cognitive function in people with hearing loss. Hence, the question arises: can audiovisual training result in better cognitive functioning in people with hearing loss than with auditory (or auditory-cognitive training)?

## Data Availability

Not applicable.
